# Tracheal Injection: Its Simplification and Its Use in Pulmonary Tuberculosis

**Published:** 1905-07-29

**Authors:** 


### TRACHEAL INJECTION: ITS SIMPLIFICATION AND ITS USE IN PULMONARY TUBERCULOSIS.

In a lecture delivered in English at the Hôtel Dieu, Dr. Mendel points out that intratracheal medication in phthisis pulmonalis has failed to become popular in consequence of the expert manipulation required for its practice, and because many of the substances administered have been irritating, and therefore provocative of cough. He claims to have devised a method by which tracheal injection can be performed without any special laryngological training, and maintains that non-irritating fluids introduced into the air-passages do not cause either reflex, cough, or suffocation. The basis of this method is a recognition of the fact that, in the absence of the act of swallowing, the œsophagus is a closed tube, so that when, in these circumstances, a small quantity of non-irritating liquid is projected against the wall of the pharynx, it runs into the only opening situated inferiorly—namely, the glottis. The fluid may be directed against the posterior wall of the pharynx (medial method), but it is more convenient to project it against the lateral wall. The syringe employed is a modification of Beehag's instrument. It is introduced through the buccal cavity with the curve of the nozzle in a horizontal plane. The outer side of the curve is then firmly applied to the base of the left faucial column by which means steadiness is secured. The nozzle thus rests horizontally in the glosso-epiglottic groove with its orifice directed to the lateral wall of the pharynx, and the fluid is now forcibly ejected so that it flows round to the posterior wall, whence it enters the glottis. Unless the syringe is steadied in the manner described the forcible ejection may be attended by movements of the nozzle and consequent irritation of the pharynx. During the manipulation the patient remains passive and breathes without effort. After the syringe is withdrawn the tongue must be kept protruded for a few seconds, as it is only under this condition that the funnel-shape of the pharynx is maintained and the entrance of fluid into the larynx is facilitated. Then the patient spits out any small quantity of liquid that may remain in the pharynx and rinses his throat and mouth. The method is thus much more simple than the usual manipulation in which fluids are introduced directly through the glottis, being delivered from a syringe the point of which is guided by the aid of a throat mirror. In Dr. Mendel's plan no laryngeal mirror is used and a head mirror is only advisable when artificial light has to be employed.

Regarding the medicines suitable for tracheal injection Dr. Mendel condemns menthol and creosote as too irritating. He employs eucalyptol (5 to 10 per cent.) and gomenol (5 to 50 per cent.) dissolved in olive oil. At first a very weak solution should be used in order to test the sensibility of the patient. Exceptionally the trachea will only tolerate, at least at the outset, a solution containing not more than one to two per cent. of the active ingredient. But this is quite unusual, and when such uncommon sensibility exists, as shown by cough, etc., it is at once soothed by an injection of pure olive oil. The treatment consisting of a syringeful of medicated fluid (three centimetres) thrice daily is continued for a month.

The results of this treatment in cases of consumption are early shown in an improvement of the respiration, at first only for a time after each injection, but later becoming permanent. The chest expansion becomes greater when the number of respirations is diminished. Consequently the defective intake of air is overcome, with the natural consequence of improved appetite and gain in weight and vitality.

In addition to these general benefits the tracheal injections remove phlegm from and cleanse the larynx; they diminish cough and expectoration; and they remove abnormal secretion from the broncho-pulmonary surfaces as is shown by the disappearance of various forms of rale. The treatment may be adopted not only in cases of phthisis pulmonalis but in cases of chronic bronchitis and other affections of the respiratory passages.

[1] Lancet, July 15, 1905.

